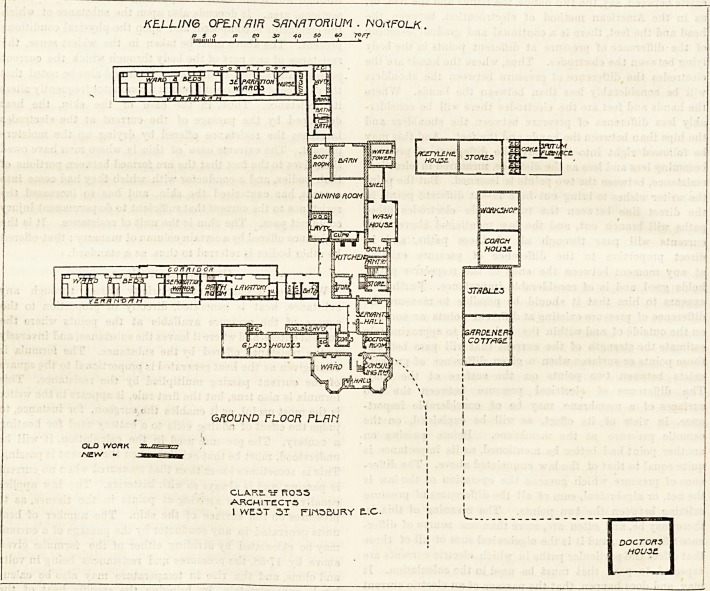# The Kelling Sanatorium, Norfolk

**Published:** 1905-06-10

**Authors:** 


### THE KELLING SANATORIUM, NORFOLK.

This sanatorium for consumptives is supported partly by voluntary contributions and partly by the patients themselves who contribute according to their means. The original building was an old farmhouse, and we gave in 1903 a short description of it as it then stood. The treatment carried out in it was found to be so successful that it was soon decided to retain the old building chiefly as an administrative centre, and to build a series of continuous cubicles for the patients. This has now been done, at least 21 new beds have been added to the available accommodation.

The site of the buildings is a quite good one. It is several hundred feet above the sea level; and it is fairly well protected from the north, east and west by a belt of trees. The sea is about five miles distant.

The old building has been considerably altered, and it now contains on the ground-floor, an entrance-hall, a consultingroom, servants' hall, storerooms, kitchen, a good dining-room, various other offices, and to the left of the entrance-hall is a small ward. The first floor contains several patients' rooms; rooms for nurses and domestic staff; bath rooms, and what is of much importance, an isolation-room. The patients' rooms in this old part of the structure can hardly be entirely satisfactory, and no doubt they will soon be disused as patients' rooms. Probably this disuse may have been already effected.

The two new pavilions run at right angles to the old building and in a westerly direction. The committee made some experiments with temporary buildings constructed of wood; and these new pavilions embody the conclusions arrived at as to the plan best suited for giving the nearest approach to sleeping in the open air, and, consequently, for the most rapid cure of consumption. One of these pavilions contains eleven beds and the other ten beds. Two and three beds are respectively in separate rooms, and the other beds are placed in double-bedded cubicles. Each cubicle has about 12 feet by 11 feet of floor space with a ceiling height of 7 feet 3 inches; and it is pointed out, we think correctly, that a greater height is unnecessary, as the whole of the pavilion is flooded with fresh air. The partitions between the cubicles are 6 feet high; there is a 5 feet opening on each side filled in with folding hatch doors so that the amount of air can be increased or diminished at will, and the idea carried out in the construction of the pavilions is that they should be merely sufficient to provide the requisite privacy, and protection from high winds and driving rains. Each pavilion is provided with a lavatory, two bathrooms, a ward-kitchen, and a nurses' bed-sitting-room. All doors and windows are made to open outwards and to fasten back flat against the walls. The pavilions are surrounded with verandahs, and the floors of these are made of concrete. The nurses can therefore visit any one of the rooms and be under cover. This part of the construction is, we believe, peculiar to the Kelling Sanatorium, and it has the enormous advantage of permitting easy communication without interfering with the cross-ventilation of the sleeping rooms, which interference is always present to some extent with the inside communicating corridors. To what extent this outside communication could be adapted to large sanatoria is another question, but in smaller ones it is correct in principle.

The walls of the pavilions are built of fireproof slabs finished on the outside with rough cast, and the roofs are covered with tiles. Although economy has been carefully studied in the construction of these pavilions, efficiency has not been sacrificed, and it is certainly worth recording that accommodation has been provided for 21 patients at a cost of £1,300, which is about £60 a bed, and includes drainage and hot and cold water supply. The buildings are lighted by acetylene gas, and the water is obtained from a deep well, from which it is pumped into a water tower, gravitating thence to all parts of the sanatorium. Many day shelters for the patients are provided in the grounds.

The architects were Messrs. Clare and Ross, of Finsbury Circus, E.C., and of Chelmsford; and the contractor was Mr. Blyth, of Foulsham, Norfolk.

## Figures and Tables

**Figure f1:**